# Communication and Handoff Errors Within Treatment-Resistant Bipolar I Disorder

**DOI:** 10.7759/cureus.23703

**Published:** 2022-03-31

**Authors:** Amna Khan, Spiro Curis, Omar Khan, Jawad Zafar

**Affiliations:** 1 Department of Behavioral Medicine and Psychiatry, Avalon University School of Medicine, Willemstad, CUW; 2 Department of Behavioral Health and Psychiatry, Avalon University School of Medicine, Willemstad, CUW; 3 Department of Behavioral Medicine and Psychiatry, Farhat Medical Clinic, Beckley, USA

**Keywords:** handoff strategies, handoff errors, noncompliance, treatment resistant, bipolar disorder

## Abstract

Bipolar I disorder includes periods of acute mania, e.g., symptoms of risk-taking behavior and impulsivity, which may result in interpersonal conflict with long-term implications. Bipolar I disorder management may be complicated by disruptions in care, both by patients and healthcare systems. We present a case of a 69-year-old male who was involuntarily committed by his wife due to inappropriate sexual behavior and delusions secondary to mania. In the emergency department, the patient appeared agitated, guarded, and with impaired cognition. His medical history included cardiac comorbidities, requiring multidisciplinary involvement.

We describe how our patient’s decompensation was contributed by a combination of noncompliance, lack of provider communication, and handoff errors. Our patient met the diagnostic criteria for treatment-resistant bipolar I disorder with mania, generalized anxiety disorder, and severe tobacco use disorder. His treatment with neuroleptics was complicated by cardiac comorbidities, indications for pacemakers, and his lack of understanding regarding the need for treatment. Our case describes a uniquely complicated admission course in part by our patient’s at-risk demographics and healthcare system shortcomings that may be more common in resource-limited facilities. We aim to integrate communication strategies for patients experiencing delusional symptoms, alongside individual and institutional strategies to mitigate systematic errors.

## Introduction

Bipolar I disorder is a mood disorder characterized by episodes of mania, hypomania, and major depression, affecting an estimated 1%-3% of adults worldwide [1]. The pathogenesis of bipolar disorder is unknown, though it may involve genetic, neurobiological, and psychosocial factors [2]. Bipolar disorder is associated with both psychiatric and medical comorbidities, including earlier onset of death [3]. Though treatment-resistant bipolar disorder is difficult to characterize due to its complex clinical presentation, course, and treatment options, it is a major health problem related to significant disability and healthcare costs [4].

Given the challenges of treatment-resistant bipolar disorder, evidence suggests that medication adherence may be determined by sociodemographic factors such as income and educational level [5]. Quality of care is especially crucial in the treatment of psychosis; patients may dispute the meaning of hallucinations or delusions and often may misunderstand, leading to poorer treatment outcomes [6]. This, in addition to inadequate handoff communication between providers and facilities, poses a significant risk to patients with treatment-resistant bipolar disorder.

This case is notable due to an intersection of the patient’s psychiatric symptoms, educational background, and systematic failures in communication, contributing to the patient’s misunderstanding and noncompliance. This case aims to identify and offer strategies, both individual and institutional, for mitigating similar shortcomings.

## Case presentation

This case report involves a current involuntary commitment and a previous involuntary commitment one month prior. This timeline is important to consider given many complications, both psychiatric and medical, that intertwine in this patient’s health outcomes.

Current presenting complaints

The patient is a 69-year-old white male with treatment-resistant bipolar I disorder who presented to the emergency department due to involuntary commitment filed by his wife. The patient’s wife reported that the patient had recently engaged in risk-taking and bizarre behavior including testing positive and recovering from COVID-19, kissing and hugging male and female strangers, and making what was perceived to be sexually inappropriate comments regarding his minor granddaughter. Interview of the patient and family revealed that the patient had not taken his prescribed neuroleptic for several weeks. The patient denied recent alcohol or illicit substance use, which was corroborated by a urine drug screen.

Upon evaluation, the patient appeared guarded though expressed delusions, grandiosity, and flight of ideas consistent with acute mania. The patient voiced paranoia regarding his wife having extramarital affairs with three African-American males in their residential complex, expressing they would enter their home at all hours. The patient, however, failed reality testing as he was unable to reason through his delusions when challenged; the family concomitantly reported the affairs as untrue. The patient also expressed feelings as though his TV was talking about him and threatening him. It is to be noted that these delusions were only present during a manic episode and were not present when the patient’s symptoms were in remission.

From the emergency department, the patient was admitted to the psychiatric unit, though he was incorrectly assigned to a psychiatrist who was not his regular treating doctor; unfortunately, this issue was not corrected for several days. This created a one-day lapse in restarting his home cariprazine for the treatment of his acute mania. In the emergency department, the patient developed bradycardia, as low as 49 bpm. After evaluation by an internist and cardiologist, the patient’s bradycardia was determined to be secondary to metoprolol succinate prescribed for the patient’s paroxysmal atrial fibrillation and chronic systolic heart failure. Metoprolol was discontinued in favor of alternate cardiac medications.

Cariprazine was later discontinued to ensure it did not exacerbate the patient's cardiac issues. In lieu of cariprazine, the patient was started on lithium and titrated to 600 mg QHS. Lithium was chosen due to the patient’s history of tolerating this well and successful treatment in the past. After initiation of lithium, the patient showed resolution of mania symptoms.

Previous admission course

The patient was involuntarily committed one month prior at the same facility, which was also filed by his wife, for acute mania symptoms secondary to medication noncompliance. The patient’s delusions similarly presented with paranoid and hypersexual components at the time. Upon this interview, the patient reported that he had not taken his prescribed neuroleptic for several days due to staying with his brother after an argument with his wife. The patient reported being functionally illiterate as he got only a seventh-grade education and is entirely relying on his wife to administer his medications for him, thereby missing these doses for this period of time.

During this admission, the patient developed bradycardia and was subsequently transferred from the psychiatric unit to the intensive care unit (ICU) for monitoring due to his known history of heart failure, atrial fibrillation, and status post percutaneous intervention. The patient’s Vraylar (cariprazine) was discontinued to limit the medication’s effect on his bradycardia to await the placement of a pacemaker.

Unfortunately, the patient left against medical advice after refusing the pacemaker placement, stating a preference for a larger facility located approximately 60 miles away. Because the patient left against medical advice, his cariprazine was not restarted prior to his departure. Although prescriptions were sent to the patient’s pharmacy, lapses in pickup and administration likely contributed to his current decompensation. During the patient’s involuntary commitment for one month after this admission, although he stated that he had been to the larger preferred facility, this was determined to be false secondary to his psychiatric condition, corroborated by medical records and history provided by the patient's wife.

## Discussion

Medication and medical noncompliance are both attributed to this patient’s multiple hospitalizations. Risk factors for nonadherence in bipolar disorder may either be intrinsic, e.g., poor insight, cognitive impairment, greater delusional or suspiciousness symptoms, or systematic, e.g., lower level of education, poor therapeutic alliance, barriers to care, or low socioeconomic status [5]. All of these risk factors, to various extents, are demonstrated by our patient.

It is understood that medical nonadherence has a negative effect on illness outcomes, leading to higher rates of hospitalizations and increased cost of the stay [5]. In 2016, the average cost per hospital stay was $11,700 in the United States; this cost was greater for patients with Medicare, averaging $13,600 per hospital stay [6]. Our patient, like others with severe and persistent mental illness, would have been an appropriate candidate for out-patient community intervention teams to avoid undue healthcare and financial burden.

Handoff strategies

Effective communication and handoff are vital for patient care and safety. Multiple studies recommend using checklists for intrahospital transport, especially for intensive care patients, to reduce complications [7]. However, checklists rely on the users’ quality of communication and adequate time management. One meta-analysis concluded the most significant problem contributing to handoff errors was poor communication between the incoming and outgoing nurses; when this was solved, other factors such as constraints posed by electronic medical records could be improved easily [7]. Still, most checklists or quality control measures are institution- and department-specific, requiring unique tailoring of what information is to be relayed or assessed.

Methods that the patients and families can adopt to overcome system failures include psychiatric advanced directives. These may include notifying a patient’s regular treating psychiatrist whenever a patient is admitted to any hospital or requiring consultation of a behavioral treatment team regardless of the reason for admission. Though advanced directives are not guaranteed safe gates, they may prevent similar errors like those mentioned during the case presentation.

Communication strategies

Providers can adopt strategies to make their language more understandable and accepted by patients, in turn benefiting therapeutic relationships. Clarification, otherwise known as repair, of providers’ speech is associated with better patient adherence to treatment [8]. Building an understanding of patients’ psychotic experiences also increases the providers’ self-repair [8]. There are two main types of repair: first, a speaker editing or reworking their speech as it occurs, and second, when a listener clarifies the speaker’s speech. Evidence supports that more patient clarification of psychiatrists’ speech was associated with better treatment adherence for six months, controlling the other factors [8].

## Conclusions

Overall, our case features a challenging admission course, alongside past history, of a patient with treatment-resistant bipolar I disorder. Our patient’s involuntary commitments can be attributed to medication noncompliance, though they are intertwined with lapses in provider communication, handoff errors, and patient misunderstanding due to education and psychiatric background. To our knowledge, our case adds to the limited literature navigating the care of patients with treatment-resistant bipolar disorder in the context of healthcare shortcomings. Further research is needed in managing and communicating with treatment-resistant bipolar disorder patients, especially in implementing institutional or departmental tools to minimize miscommunication and handoff errors.

## References

[1] Pedersen CB, Mors O, Bertelsen A (2014). A comprehensive nationwide study of the incidence rate and lifetime risk for treated mental disorders. JAMA Psychiatry.

[2] (2022). Bipolar disorder in adults: epidemiology and pathogenesis. https://www.uptodate.com/contents/bipolar-disorder-in-adults-epidemiology-and-pathogenesis.

[3] Crump C, Sundquist K, Winkleby MA, Sundquist J (2013). Comorbidities and mortality in bipolar disorder: a Swedish national cohort study. JAMA Psychiatry.

[4] Fountoulakis KN, Yatham LN, Grunze H (2020). The CINP guidelines on the definition and evidence-based interventions for treatment-resistant bipolar disorder. Int J Neuropsychopharmacol.

[5] García S, Martínez-Cengotitabengoa M, López-Zurbano S, Zorrilla I, López P, Vieta E, González-Pinto A (2016). Adherence to antipsychotic medication in bipolar disorder and schizophrenic patients: a systematic review. J Clin Psychopharmacol.

[6] (2022). National inpatient hospital costs: the most expensive conditions by payer, 2017. https://www.hcup-us.ahrq.gov/reports/statbriefs/sb261-Most-Expensive-Hospital-Conditions-2017.jsp.

[7] Raeisi A, Rarani MA, Soltani F (2019). Challenges of patient handover process in healthcare services: a systematic review. J Educ Health Promot.

[8] McCabe R, Healey PG (2018). Miscommunication in doctor-patient communication. Top Cogn Sci.

